# Supplementary material for: Genetic risk and immune dysregulation of classic Hodgkin lymphoma transformation of chronic lymphocytic leukemia/small lymphocytic lymphoma: a multicentric study
Source: medRxiv. 2026 May 20:2026.05.11.26352584. Preprint. [Version 1] doi: 10.64898/2026.05.11.26352584 (PMC13228784; doi:10.64898/2026.05.11.26352584)
Supplement: Supplement 1 [file media-1.pdf]

Supplementary table 1:

Patient demographics, clinicopathologic characteristics, and ancillary testing

		Demographics					Ancillary tests			IHC and ISH results									Treatments and outcomes		
**ID**	**CLL/SLL type**	**Sex**	**Age range at CLL/SLL dx**	**Age range at CHL-RT dx**	**HRS cell % in CLL/SLL**	**Months between CLL/SLL and CHL**	**NGS tested**	**FISH tested**	**mIF tested**	**EBER**	**CD30**	**CD15**	**PD-L1**	**PD-L2**	**MHC-I**	**B2M**	**MHC-II**	**CIITA**	**CLL treatment**	**CHL treatment**	**Outcome**
**1**	Type 1	M	76-80	76-80	<5%	0	Yes	No	No	-	+	+	-	-	+	-	-	+	NA	NA	Alive
**2**	Type 1	F	61-65	76-80	<5%	176.4	Yes	No	Yes	+	+	-	NA	NA	NA	+	NA	+	PCR >> ibrutinib	AVD + ibrutinib >> V + OB	Alive
**3**	Type 1	M	71-75	76-80	<5%	70.5	No	No	No	-	+	+	NA	NA	NA	NA	NA	NA	NA	R-CHOP >> R + benda >> ibrutinib >> R-GCVP >> palliative RT	Deceased
**4**	Type 1	F	81-85	86-90	~5%	66.3	No	No	No	NA	+	-	NA	NA	NA	NA	NA	NA	ibrutinib >> observation	NA	Alive
**5**	Type 1	M	NA	71-75	<5%	NA	No	No	No	-	+	+	NA	NA	NA	NA	NA	NA	NA	NA	Alive
**6**	Type 1	M	NA	86-90	<5%	NA	No	No	No	-	+	-	NA	NA	NA	NA	NA	NA	NA	NA	Alive
**7**	Type 1	M	41-45	41-45	<5%	0	No	No	No	NA	+	+	NA	NA	NA	NA	NA	NA	NA	ABVD	Alive
**8**	Type 1	M	56-60	56-60	<5%	0	Yes	No	No	+	+	+	+	-	NA	-	-	+	NA	NA	NA
**9**	Type 1	F	86-90	86-90	<5%	0	Yes	No	No	-	+	+	-	NA	NA	NA	-	NA	NA	NA	NA
**10**	Type 1	M	NA	51-55	~5%	NA	Yes	No	No	+	+	+	+	-	+	+	-	+	NA	NA	NA
**11**	Type 1	M	56-60	61-65	<5%	109.6	Yes	No	No	+	+	-	-	-	NA	-	NA	+	NA	NA	NA
**12**	Type 1	M	56-60	56-60	<5%	48.7	Yes	No	No	+	+	+	+	-	+	+	+	-	NA	NA	NA
**13**	Type 1	M	66-70	66-70	<5%	0	Yes	No	Yes	+	+	-	-	-	+	-	+	+	NA	NA	NA
**14**	Type 1	F	NA	66-70	~5%	NA	Yes	Yes	No	-	+	+	-	-	+	+	+	+	NA	NA	NA
**15**	Type 1	M	NA	61-65	<5%	NA	Yes	No	No	-	+	+	+	-	+	+	+	+	NA	NA	NA
**16**	Type 1	M	NA	61-65	10-20%	NA	Yes	No	No	-	+	+	-	NA	NA	+	+	NA	NA	NA	NA
**17**	Type 1	F	66-70	71-75	<5%	104.3	No	No	No	+	+	-	NA	NA	NA	NA	NA	NA	NA	NA	Alive
**18**	Type 1	M	56-60	66-70	<5%	70.7	No	No	No	+	+	-	NA	NA	NA	NA	NA	NA	NA	NA	Deceased
**19**	Type 1	F	61-65	66-70	<5%	59.2	Yes	No	No	+	+	+	+	-	+	+	-	+	NA	NA	Deceased
**20**	Type 1	M	66-70	71-75	~5%	150.3	Yes	Yes	Yes	+	+	-	-	-	+	-	+	+	NA	NA	Deceased
**21**	Type 1	M	66-70	71-75	<5%	75.7	Yes	No	No	+	+	+	+	-	-	+	+	+	NA	NA	Deceased
**22**	Type 1	M	81-85	81-85	<5%	34.8	Yes	No	No	+	+	-	NA	NA	NA	NA	NA	NA	NA	NA	Deceased
**23**	Type 1	M	46-50	81-85	<5%	420.4	No	No	No	+	+	-	NA	NA	NA	NA	NA	NA	NA	NA	Deceased
**24**	Type 1	M	71-75	81-85	<5%	126.4	Yes	No	No	-	+	+	NA	NA	NA	NA	NA	NA	NA	NA	Deceased
**25**	Type 1	M	61-65	71-75	<5%	104.5	Yes	Yes	No	NA	+	+	+	-	-	+	+	+	NA	NA	Deceased
**26**	Type 1	M	56-60	61-65	<5%	61.4	Yes	Yes	No	+	+	+	+	-	+	-	-	+	NA	NA	Deceased
**27**	Type 1	M	71-75	76-80	<5%	45.2	No		No	+	+	-	NA	NA	NA	NA	NA	NA	NA	NA	Alive
**28**	Type 2	M	41-45	46-50	~10%	58.8	Yes	Yes	No	-	+	+	NA	NA	NA	NA	NA	+	PCR >> CAR-T >> RT >> ibrutinib	ABVD >> R + idelalisib >> GCV >> OB >> benda + OB >> SCT >> pembrolizumab	Alive

**29 #**	Type 2	M	61-65	61-65	<5%	0	Yes	No	No	+	+	-	NA	NA	NA	NA	NA	+	NA	R-EPOCH + V >> SCT	Alive
**30 ***	Type 2	M	76-80	76-80	~5%	0.25	Yes	Yes	No	-	+	-	+	NA	NA	+	-	+	NA	NA	Alive
**31**	Type 2	M	61-65	76-80	<5%	190.9	No	No	No	+	+	+	NA	NA	NA	NA	NA	NA	NA	NA	Deceased
**32**	Type 2	M	61-65	61-65	5-10%	0	No	No	No	NA	+	+	NA	NA	NA	NA	NA	NA	NA	ABVD >> AVD	Alive
**33**	Type 2	M	46-50	46-50	5-10%	0	No	No	No	+	+	-	+	-	+	-	+	+	NA	NA	Deceased
**34**	Type 2	M	66-70	66-70	<5%	50.3	No	No	No	NA	+	+	+	-	+	+	-	+	acalabrutinib	BV-AVD >> nivolumab + AD >> OB + V	Alive
**35**	Type 2	M	61-65	61-65	5-10%	0	No	No	No	NA	+	+	NA	NA	NA	NA	NA	NA	NA	BV >> AVD >> BV	Alive
**36**	Type 2	M	71-75	76-80	<5%	66.5	Yes	No	No	NA	+	-	+	-	+	+	+	+	observation	BV >> AVD	Alive
**37**	Type 2	M	71-75	71-75	<5%	0	Yes	No	Yes	-	+	-	NA	NA	NA	NA	NA	+	NA	NA	Deceased
**38**	Type 2	F	61-65	61-65	<5%	0	No	No	No	+	+	+	NA	NA	NA	NA	NA	NA	NA	NA	Alive
**39**	Type 2	M	51-55	56-60	<5%	45.1	No	No	No	NA	+	-	+	-	+	NA	+	+	ibrutinib >> R	BV + AVD	Alive
**40**	Type 2	M	36-40	36-40	<5%	0	No	No	No	+	+	+	NA	NA	NA	NA	NA	NA	NA	ABVD >> RT	Alive
**41**	Type 2	F	61-65	66-70	<5%	56.7	No	No	No	NA	+	+	NA	NA	NA	NA	NA	NA	observation >> FCR >> BR >> ibrutinib	ABVD	Deceased
**42**	Type 2	M	86-90	86-90	<5%	12.1	No	No	No	-	+	+	NA	NA	NA	NA	NA	NA	NA	COPP >> BV >> ibrutinib	Deceased
**43**	Type 2	M	36-40	36-40	<5%	6.8	No	No	No	+	+	-	NA	NA	NA	NA	NA	NA	FCR	ABVD	Alive
**44**	Type 2	M	56-60	81-85	<5%	265.4	No	No	No	NA	+	+	NA	NA	NA	NA	NA	NA	observation >> bleomycin + CHOP >> R	R-CEPP >> BV >> acalabrutinib	Deceased
**45**	Type 2	M	61-65	66-70	<5%	73	No	No	No	NA	+	+	NA	NA	NA	NA	NA	NA	observation	ABVD >> GVD >> SCT >> ibrutinib >> V	Deceased
**46**	Type 2	F	NA	36-40	5-10%	NA	No	No	No	NA	+	+	NA	NA	NA	NA	NA	NA	NA	NA	Alive
**47**	Type 2	M	71-75	71-75	<5%	2.8	No	No	No	-	+	-	NA	NA	NA	NA	NA	NA	NA	R-CHOP >> RT	Alive
**48**	Type 2	M	66-70	71-75	<5%	36.5	No	No	No	NA	+	-	NA	NA	NA	NA	NA	NA	V + OB	BV >> AVD >> BV	Alive
**49**	Type 2	F	56-60	71-75	5-10%	166.3	No	No	No	+	+	+	NA	NA	NA	NA	NA	NA	observation >> ibrutinib >> V	benda + ibrutinib >> AVD >> BV	Alive
**50**	Type 2	M	66-70	71-75	~5%	36.5	Yes	No	No	-	+	+	+	-	NA	+	-	NA	NA	NA	NA
**51**	Type 2	F	66-70	66-70	~10%	36.5	Yes	Yes	No	-	+	+	+	-	+	-	-	+	NA	NA	NA
**52**	Type 2	M	66-70	66-70	<5%	0	Yes	No	No	+	+	+	+	-	+	+	+	-	NA	NA	NA
**53**	Type 2	M	71-75	71-75	~10%	0	Yes	Yes	No	-	+	+	+	-	-	-	-	+	NA	NA	NA
**54**	Type 2	M	NA	66-70	<5%	NA	Yes	No	No	+	+	+	+	NA	NA	+	-	+	NA	NA	NA
**55**	Type 2	F	NA	81-85	10-20%	NA	Yes	Yes	Yes	+	+	+	+	-	-	+	-	+	NA	NA	NA
**56**	Type 2	M	66-70	76-80	<5%	97.4	No	No	No	-	+	+	+	-	+	+	-	+	NA	NA	NA
**57**	Type 2	F	NA	61-65	<5%	NA	No	No	No	NA	+	+	+	-	-	-	-	+	NA	NA	NA
**58**	Type 2	M	66-70	71-75	<5%	24.4	Yes	No	No	+	+	+	+	-	-	NA	-	+	NA	NA	NA
**59**	Type 2	F	NA	71-75	20-30%	NA	Yes	Yes	No	+	+	+	+	NA	NA	-	+	NA	NA	NA	NA
**60**	Type 2	F	46-50	46-50	<5%	0	No	No	No	+	+	+	+	-	+	-	-	+	NA	NA	NA
**61**	Type 2	F	NA	56-60	<5%	NA	No	No	No	+	+	-	+	NA	NA	-	+	+	NA	NA	NA
**62**	Type 2	M	NA	71-75	~5%	NA	Yes	No	No	+	+	-	+	-	+	-	+	+	NA	NA	NA
**63 ***	Type 2	M	56-60	66-70	<5%	66.8	Yes	Yes	No	+	+	+	+	NA	NA	-	+	-	NA	NA	Alive
**64**	Type 2	M	46-50	51-55	<5%	58.5	Yes	No	No	+	+	+	+	-	+	+	-	+	NA	NA	Deceased
**65**	Type 2	F	66-70	71-75	~10%	25.2	Yes	Yes	Yes	NA	+	+	+	-	+	+	+	+	NA	NA	Deceased

**66**	Type 2	F	61-65	71-75	<5%	134.2	No	No	No	+	+	+	NA	NA	NA	NA	NA	NA	NA	NA	Deceased
**67**	Type 2	M	61-65	61-65	<5%	0.1	No	No	No	+	+	-	NA	NA	NA	NA	NA	NA	NA	NA	Deceased
**68**	Type 2	M	56-60	66-70	10-20%	117.5	Yes	No	No	+	+	+	+	+	-	-	+	+	NA	NA	Deceased
**69**	Type 2	M	61-65	71-75	<5%	112.2	Yes	No	No	-	+	+	+	+	+	+	+	+	NA	NA	Deceased
**70**	Type 2	M	71-75	81-85	<5%	117	No	No	No	+	+	+	NA	NA	NA	NA	NA	NA	NA	NA	Deceased
**71 ***	Type 2	M	71-75	76-80	<5%	42.4	Yes	No	No	-	+	-	+	NA	-	+	+	+	NA	NA	Deceased
**72**	Type 2	M	41-45	51-55	<5%	138.2	No	No	No	+	+	+	NA	NA	NA	NA	NA	NA	NA	NA	Deceased
**73**	Type 2	F	66-70	71-75	<5%	79.1	No	No	No	-	+	+	NA	NA	NA	NA	NA	NA	NA	NA	Alive
**74**	Type 2	M	56-60	61-65	~5%	69.7	Yes	No	No	+	+	+	+	NA	NA	+	-	+	NA	NA	Deceased
**75**	Type 2	M	61-65	71-75	<5%	74.9	No	No	No	-	+	+	NA	NA	NA	NA	NA	NA	NA	NA	Deceased
**76**	Type 2	M	66-70	71-75	5-10%	33.9	No	No	No	-	+	+	+	-	+	+	+	+	NA	NA	Deceased
**77**	Type 2	F	66-70	86-90	<5%	211	No	No	No	+	+	-	+	-	+	+	+	+	NA	NA	Deceased

Abbreviations: **ABVD**: doxorubicin, bleomycin, vinblastine, dacarbazine. **AD**: doxorubicin and dacarbazine. **AVD**: doxorubicin, vinblastine, dacarbazine. **Benda**: bendamustine. **BR**: bendamustine and rituximab. **BV**: brentuximab vedotin. **CAR-T**: chimeric antigen receptor-T cell therapy. **CEPP**: cyclophosphamide, etoposide, procarbazine, and prednisone. **CHOP**: cyclophosphamide, doxorubicin, vincristine, and prednisone. **COPP**: cyclophosphamide, vincristine, procarbazine hydrochloride, and prednisone. **EPOCH**: etoposide phosphate, prednisone, vincristine, cyclophosphamide, and doxorubicin. **FCR**: fludarabine, cyclophosphamide, and rituximab. **GCV**: gemcitabine, cyclophosphamide, vincristine. **GCVP**: gemcitabine, cyclophosphamide, vincristine, prednisolone. **GVD**: gemcitabine, vincristine, and doxorubicin. **IF**: immunofluorescence. **IHC**: Immunohistochemistry. **ISH**: In-situ hybridization. **mIF**: multiplex immunofluorescence. **OB**: obinutuzumab. **PCR**: pentostatin, cyclophosphamide, and rituximab. **R**: rituximab. **RT**: radiation therapy. **SCT**: stem cell transplantation. **V**: venetoclax.

Note: # For this patient, two samples—one with type 1 and another with type 2 CLL/SLL—were submitted for NGS studies.

* (NGS) studies were conducted on macrodissected CLL/SLL-enriched and CHL-enriched areas separately for each of these patients.

Supplementary table 2:

Gene list for MSK IMPACT-Heme

ABL1	*ACTG1*	*AKT1*	*AKT2*	*AKT3*	*ALK*	*ALOX12B*	*AMER1*	*APC*	*AR*
ARAF	*ARHGEF28*	*ARID1A*	*ARID1B*	*ARID2*	*ARID3A*	*ARID3B*	*ARID3C*	*ARID4A*	*ARID4B*
ARID5A	*ARID5B*	*ASXL1*	*ASXL2*	*ATM*	*ATP6AP1*	*ATP6V1B2*	*ATR*	*ATRX*	*ATXN2*
AURKA	*AURKB*	*AXIN1*	*AXL*	*B2M*	*BACH2*	*BAP1*	*BARD1*	*BCL10*	*BCL11B*
BCL2	*BCL6*	*BCOR*	*BCORL1*	*BCR*	*BIRC3*	*BLM*	*BRAF*	*BRCA1*	*BRCA2*
BRD4	*BRIP1*	*BTG1*	*BTK*	*CALR*	*CARD11*	*CASP8*	*CBFB*	*CBL*	*CCND1*
CCND2	*CCND3*	*CCNE1*	*CD274*	*CD28*	*CD58*	*CD79A*	*CD79B*	*CDC73*	*CDH1*
CDK12	*CDK4*	*CDK6*	*CDK8*	*CDKN1B*	*CDKN2A*	*CDKN2B*	*CDKN2C*	*CEBPA*	*CHEK1*
CHEK2	*CIC*	*CIITA*	*CRBN*	*CREBBP*	*CRKL*	*CRLF2*	*CSF1R*	*CSF3R*	*CTCF*
CTNNB1	*CUX1*	*CXCR4*	*CYLD*	*DAXX*	*DDR2*	*DDX3X*	*DIS3*	*DNMT3A*	*DOT1L*
DTX1	*DUSP22*	*EED*	*EGFR*	*EGR1*	*EP300*	*EP400*	*EPHA3*	*EPHA5*	*EPHA7*
EPHB1	*ERBB2*	*ERBB3*	*ERBB4*	*ERG*	*ESCO2*	*ESR1*	*ETNK1*	*ETV6*	*EZH2*
FANCA	*FANCC*	*FANCD2*	*FAS*	*FAT1*	*FBXO11*	*FBXW7*	*FGF19*	*FGF3*	*FGF4*
FGFR1	*FGFR2*	*FGFR3*	*FGFR4*	*FLCN*	*FLT1*	*FLT3*	*FLT4*	*FOXL2*	*FOXO1*
FOXP1	*FURIN*	*FYN*	*GATA1*	*GATA2*	*GATA3*	*GNA11*	*GNA12*	*GNA13*	*GNAQ*
GNAS	*GNB1*	*GRIN2A*	*GSK3B*	*H1-2*	*H2BC5*	*H3C2*	*H3C8*	*HDAC1*	*HDAC4*
HDAC7	*HGF*	*HIF1A*	*HIST1H1B*	*HIST1H1D*	*HIST1H1E*	*HIST1H2AC*	*HIST1H2AG*	*HIST1H2AL*	*HIST1H2AM*
HIST1H2BC	*HIST1H2BG*	*HIST1H2BJ*	*HIST1H2BK*	*HIST1H2BO*	*HLA-A*	*HNF1A*	*HRAS*	*ID3*	*IDH1*
IDH2	*IGF1*	*IGF1R*	*IGF2*	*IKBKE*	*IKZF1*	*IKZF3*	*IL7R*	*INPP4B*	*IRF1*
IRF4	*IRF8*	*IRS2*	*JAK1*	*JAK2*	*JAK3*	*JARID2*	*JUN*	*KDM5A*	*KDM5C*
KDM6A	*KDR*	*KEAP1*	*KIT*	*KMT2A*	*KMT2B*	*KMT2C*	*KMT2D*	*KMT5A*	*KRAS*
KSR2	*LCK*	*LMO1*	*LTB*	*MALT1*	*MAP2K1*	*MAP2K2*	*MAP2K4*	*MAP3K1*	*MAP3K13*
MAP3K14	*MAPK1*	*MAPK3*	*MCL1*	*MDM2*	*MDM4*	*MED12*	*MEF2B*	*MEN1*	*MET*
MGA	*MGAM*	*MITF*	*MLH1*	*MOB3B*	*MPEG1*	*MPL*	*MRE11*	*MSH2*	*MSH6*
MTOR	*MUTYH*	*MYC*	*MYCL*	*MYCN*	*MYD88*	*NBN*	*NCOR1*	*NCOR2*	*NCSTN*
NF1	*NF2*	*NFE2*	*NFE2L2*	*NKX2-1*	*NOTCH1*	*NOTCH2*	*NOTCH3*	*NOTCH4*	*NPM1*
NRAS	*NSD1*	*NT5C2*	*NTRK1*	*NTRK2*	*NTRK3*	*P2RY8*	*PAK7*	*PALB2*	*PARP1*
PAX5	*PBRM1*	*PCBP1*	*PDCD1*	*PDGFRA*	*PDGFRB*	*PDPK1*	*PDS5B*	*PHF6*	*PIGA*
PIK3C2G	*PIK3C3*	*PIK3CA*	*PIK3CG*	*PIK3R1*	*PIK3R2*	*PIM1*	*PLCG1*	*PLCG2*	*PMS2*
PNRC1	*POT1*	*PPP2R1A*	*PRDM1*	*PRKAR1A*	*PTCH1*	*PTEN*	*PTPN1*	*PTPN11*	*PTPN2*
RAD21	*RAD50*	*RAD51*	*RAD51B*	*RAD51C*	*RAD51D*	*RAD52*	*RAD54L*	*RAF1*	*RARA*
RB1	*REL*	*RET*	*RHOA*	*RICTOR*	*RNF43*	*ROBO1*	*ROS1*	*RPTOR*	*RRAGC*
RTEL1	*RUNX1*	*RUNX1T1*	*SAMHD1*	*SDHA*	*SDHB*	*SDHC*	*SDHD*	*SETBP1*	*SETD1A*
SETD1B	*SETD2*	*SETD3*	*SETD4*	*SETD5*	*SETD6*	*SETD7*	*SETDB1*	*SETDB2*	*SF3B1*
SGK1	*SH2B3*	*SMAD2*	*SMAD4*	*SMARCA4*	*SMARCB1*	*SMARCD1*	*SMC1A*	*SMC3*	*SMG1*
SMO	*SOCS1*	*SOX2*	*SP140*	*SPEN*	*SPOP*	*SRC*	*SRSF2*	*STAG1*	*STAG2*
STAT3	*STAT5A*	*STAT5B*	*STAT6*	*STK11*	*SUFU*	*SUZ12*	*SYK*	*TBL1XR1*	*TBX3*
TENT5C	*TERT*	*TET1*	*TET2*	*TET3*	*TGFBR2*	*TNFAIP3*	*TNFRSF14*	*TOP1*	*TP53*
TP53	*TP63*	*TRAF2*	*TRAF3*	*TRAF5*	*TSC1*	*TSC2*	*TSHR*	*TYK2*	*U2AF1*
U2AF2	*UBR5*	*VAV1*	*VAV2*	*VHL*	*WHSC1*	*WT1*	*XBP1*	*XPO1*	*ZRSR2*
